# COVID-19 vaccination exacerbates ex vivo IL-6 release from isolated PBMCs

**DOI:** 10.1038/s41598-023-35731-2

**Published:** 2023-06-12

**Authors:** Dominik Langgartner, Raphael Winkler, Jonas Brunner-Weisser, Nicolas Rohleder, Marc N. Jarczok, Harald Gündel, Katja Weimer, Stefan O. Reber

**Affiliations:** 1 Laboratory for Molecular Psychosomatics, Department of Psychosomatic Medicine and Psychotherapy, Ulm University Medical Center, 89081 Ulm, Germany; 2 Department of Psychosomatic Medicine and Psychotherapy, Ulm University Medical Center, 89081 Ulm, Germany; 3 Department of Psychology, Chair of Health Psychology, Friedrich-Alexander-Universität Erlangen-Nürnberg, 91052 Erlangen, Germany

**Keywords:** Immunology, Adaptive immunity, Inflammation, Innate immunity, Vaccines

## Abstract

Ex vivo culturing of isolated PBMCs from individuals vaccinated with the coronavirus disease 2019 (COVID-19) vaccine BNT162b1 revealed a pronounced T cell response in the presence of the receptor binding domain (RBD) of the severe acute respiratory syndrome coronavirus 2 (SARS-CoV-2) spike protein. The latter was 10-fold more pronounced than the ex vivo response of PBMCs from the same individuals to other common pathogen T cell epitope pools, suggesting COVID-19 vaccination to induce RBD-specific T cell responses and not to facilitate T cell (re)activity in general. In the current study we investigated whether COVID-19 vaccination long-lastingly affects plasma interleukin (IL)-6 concentrations, complete blood counts, ex vivo IL-6 and IL-10 secretion of PBMCs cultured under basal conditions or in the presence of concanavalin (Con) A and lipopolysaccharide (LPS), salivary cortisol and α-amylase, mean arterial pressure (MAP), heart rate (HR) as well as mental and physical health status. The study was initially designed to investigate whether the presence vs. absence of own pets during urban upbringing has protective effects against psychosocial stress-induced immune activation during adulthood. However, as COVID-19 vaccines were approved while the study was ongoing and as, therefore, both vaccinated and non-vaccinated individuals have been recruited, we were able to stratify our data set with respect to the COVID-19 vaccination status and to assess the long-lasting effects of COVID-19 vaccination on physiological immunological, cardiovascular and psychosomatic health parameters. This data is presented in the current study. We show that isolated PBMCs from individuals vaccinated against COVID-19 show a ~ 600-fold increase in basal and a ~ 6000-fold increase in ConA-induced proinflammatory IL-6 secretion, and a ~ 2-fold increase in basal and ConA-induced antiinflammatory IL-10 secretion, both in comparison with non-vaccinated individuals. In contrast, LPS-induced ex vivo IL-6 and IL-10 secretions were not affected by vaccination status, as were plasma IL-6 concentrations, complete blood counts, salivary cortisol and α-amylase, cardiovascular measures and psychosomatic health. In summary, our findings are of relevance for many clinical studies ran before/during the pandemic, clearly indicating that consideration of participants’ vaccination status is critical, at least when assessing ex vivo PBMC functionality.

## Introduction

A growing number of questionable research practices over the last decades, including the exploration of multiple dependent variables or covariates but only reporting these when yielding significant effects, contributed to the fact that many scientific studies are difficult or impossible to reproduce^1^. As a consequence of the growing awareness of this problem, the term “replication crisis” has been coined in the early 2010s^2^, which today (i.e., 10/2022) generates over 1000 hits on PubMed.

One novel environmental covariate that might be of relevance for all clinical studies, especially for those enrolling participants/patients both before and during the coronavirus disease 2019 (COVID-19) pandemic, is the severe acute respiratory syndrome coronavirus 2 (SARS-CoV-2) vaccination status. Preclinical and clinical studies convincingly showed that adequate T cell responses, besides neutralizing antibody responses, represent a critical component of the protective SARS-CoV-2 antiviral immunity induced by COVID-19 vaccination, especially with respect to the prevention of severe COVID-19 symptomatology^3–5^. In detail, ex vivo culturing of isolated blood mononuclear cells (PBMCs) from individuals administered with the COVID-19 vaccine BNT162b1 revealed a pronounced T cell response in the presence of the receptor binding domain (RBD) of the SARS-CoV-2 spike protein. The latter was 10-fold more pronounced than the ex vivo response of PBMCs from the same individuals to other common pathogen T cell epitope pools^6^, suggesting, at least at the first glance, COVID-19 vaccination to induce SARS-CoV-2 spike protein-specific T cell responses and not to facilitate T cell (re)activity in general. Of note, a selective and long-lasting SARS-CoV-2 spike protein specific effect has been also reported in macrophages derived from COVID-19 patients and convalescents. In detail, while ex vivo lipopolysaccharide (LPS)/nigericin treatment led to the secretion of interleukin (IL)-1β from both COVID-19 patient-derived macrophages and macrophages from SARS-CoV-2 naive individuals, which was more pronounced in the former, SARS-CoV-2 spike protein/nigericin treatment selectively induced secretion of IL-1β in patient-derived cells only^7^. In contrast, inflammasome independent cytokine secretion (i.e., tumor necrosis factor, TNF) from ex vivo cultured macrophages in response to LPS was not different between the groups^7^.

The present clinical study was originally designed to assess whether the presence vs. absence of own pets during urban upbringing has protective effects against psychosocial stress-induced immune activation during adulthood. Therefore, we recruited young, physically and emotionally healthy male participants raised in a city with more than 40,000 residents either in the absence or presence of own pets (i.e., at least one dog or cat) during at least five out of the first fifteen years of life, respectively. Participants were individually exposed to the Trier social stress test (TSST)^8^, and before and after the TSST, blood was drawn for assessment of plasma interleukin (IL)-6 concentrations, complete blood count analysis as well as isolation and ex vivo culturing of PBMCs, amongst others. Moreover, systolic and diastolic blood pressure for calculation of mean arterial pressure (MAP) and heart rate (HR) were repeatedly assessed, and mental and physical health status, early life and perceived life stress, and subjective strain induced by TSST exposure were assessed using standardized and validated questionnaires. The manuscript reporting these data is currently in preparation (Langgartner, Weimer et al., unpublished). Given that participants were enrolled between July 2020 and April 2022, with the COVID-19 pandemic and respective vaccinations starting in 2019/20 and 2020/21, respectively, some of our participants from both experimental groups were vaccinated against COVID-19 (VACs), while others were not (noVACs). Therefore, we assessed in the current study whether SARS-CoV-2 vaccinations, independent of the TSST exposure, affect the cardiovascular and systemic in vivo immune status, ex vivo cytokine release from isolated PBMCs under basal conditions, but also in the presence of concanavalin (Con) A and LPS, salivary α-amylase and plasma cortisol concentrations as well as the individual mental and physical health status of all participants.

## Results

### Vaccination status (Fig. 1, Tab. S2)

**Figure 1:**
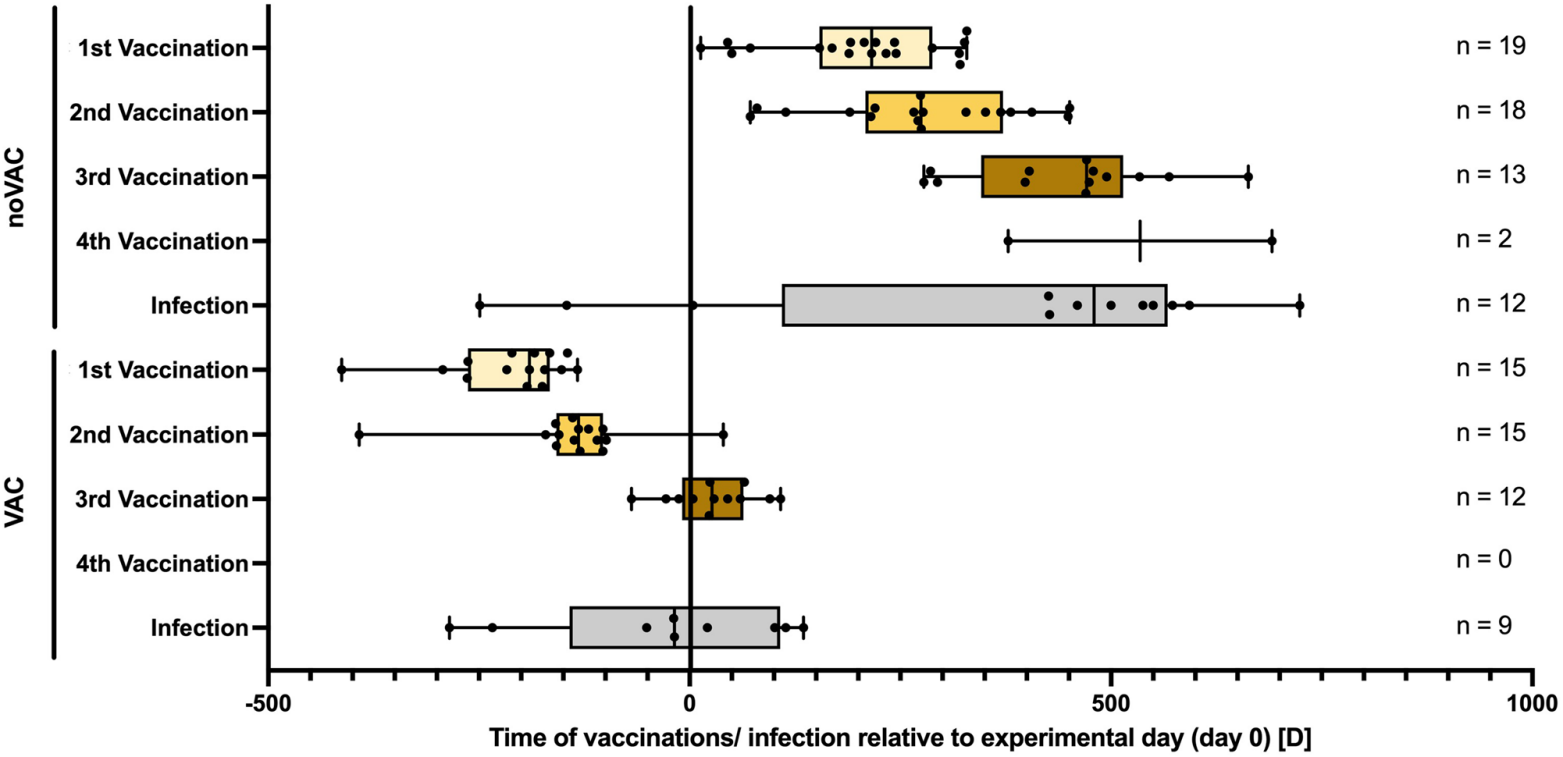
Visualization of the time periods between individual COVID-19 vaccinations or infection and the experimental day (i.e., day 0) for each participant of the COVID-19-unvaccinated (noVAC) and COVID-19-vaccinated (VAC) group. Abbreviations: D, day; Vac, vaccination. Data are presented as boxplots including the median (line), the 25th and 75th percentile, as well as the minimum/maximum value (whiskers) and the individual datapoints.

Retrospective assessment of the COVID-19 vaccination status of all included 39 participants revealed that the first 24 participants (own pet: *n* = 8; no own pet: *n* = 16) enrolled before the 8th of May 2021 were not vaccinated against COVID-19, while the 15 participants (own pet: *n* = 11; no own pet: *n* = 4) enrolled after this date were vaccinated against COVID-19 at least once, with the most recent vaccination occurring minimum two weeks prior to the experimental day (Fig. 1; Tab. S2). One out of the 15 participants in the VAC group was vaccinated once, eleven were vaccinated twice and another three already three times prior to participating in the current study (Fig. 1; Tab. S2). As the current study was statistically underpowered to stratifying the participants according to the types of vaccines received, we subclassified them simply into a VAC (i.e., receiving at least one vaccination) and a noVAC group, to assess the physiological, immunological and affective impact of COVID-19 vaccinations in healthy male individuals. Moreover, two participants in the noVAC group as well as five participants in the VAC group were infected with SARS CoV-2 prior to the experimental day, respectively (Fig. 1). Of note, four participants in the noVAC group could not be contacted retrospectively and, thus, are not visualized in Fig. 1. However, as these were enrolled in the study prior to the release of the vaccines, they have been assigned to the noVAC group. One participant in the noVAC group was still unvaccinated at the time point of manuscript publication and is therefore not visualized in Fig. 1.

### Socioeconomic parameters (Table S1)

VAC and noVAC participants differed in the “professional group (*P* = 0.040)”, “professional situation (*P* = 0.042)” and “high income” (i.e., more than 1500 € net income per month; *P* = 0.036), with more participants in the noVAC vs. VAC group reporting “high income” and “full-time employment”, while more participants in the VAC vs. noVAC group reported “in training”. Moreover, the VAC and noVAC group further differed significantly (Table S1) with respect to animal (i.e. pet and/or farm animals) contact during early life (i.e., until the age of fifteen; *P* = 0.028) and also at present (*P* = 0.004).

### Immune parameters (Figs. 2 and 3A–F)

**Figure 2:**
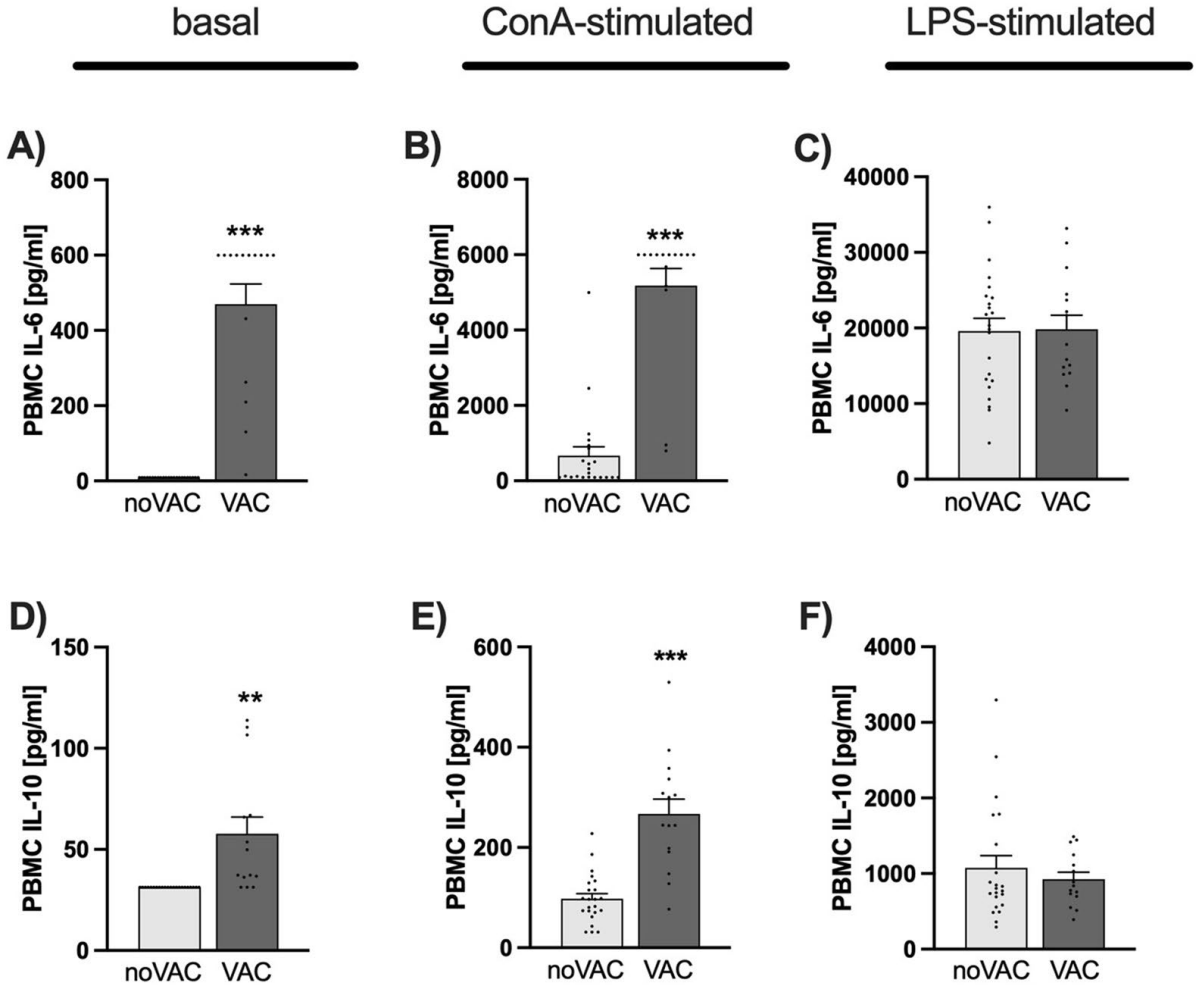
Effects of COVID-19 vaccination (VAC) on ex vivo immune readouts. (**A–C**) Ex vivo interleukin (IL)-6 secretion [pg/ml] from isolated PBMCs under basal conditions (**A**), in the presence of the T cell-specific mitogen concanavalin A (ConA; **B**) and in the presence of bacterial lipopolysaccharide (LPS; **C**). (**D–F**) Ex vivo IL-10 secretion [pg/ml] from isolated PBMCs under basal conditions (**D**), in the presence of ConA (**E**) and in the presence of LPS (**F**). Data are presented as mean + SEM including individual values. **P ≤ 0.01, ***P ≤ 0.001 versus respective participants not vaccinated against SARS-CoV-2 (noVAC).

**Figure 3:**
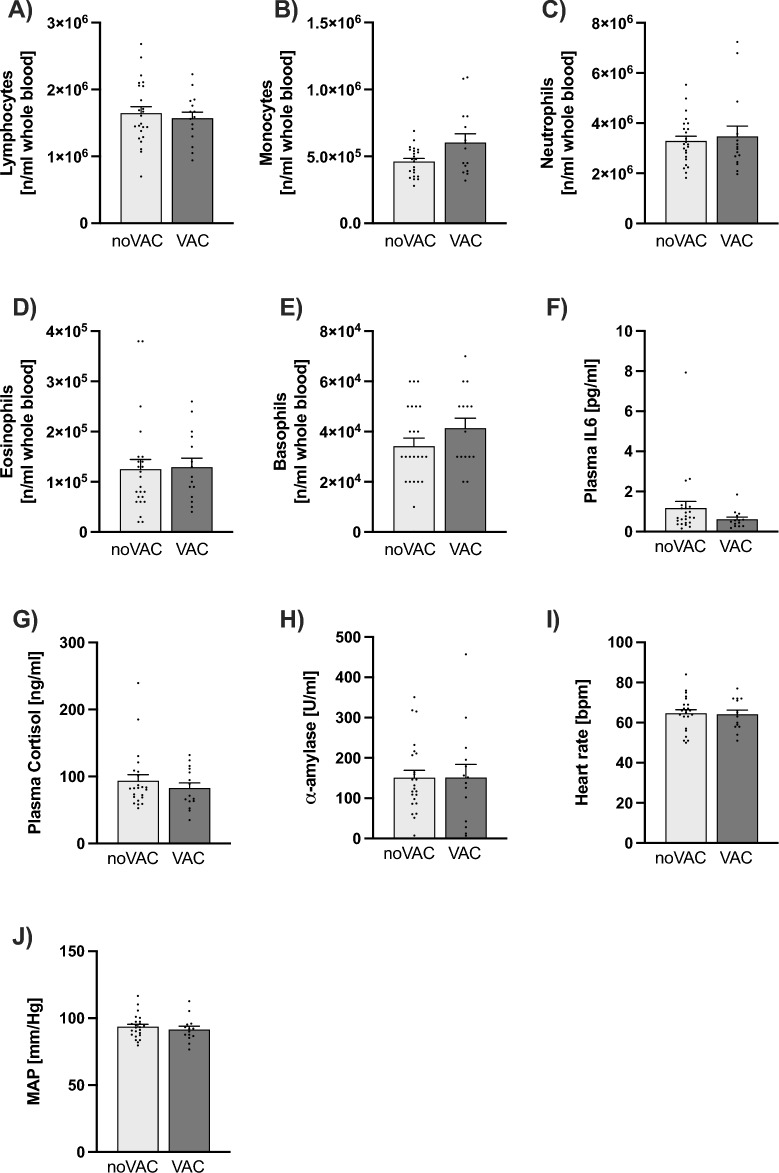
Effects of COVID-19 vaccination (VAC) on in vivo immune readouts, basal HPA axis, SNS and cardiovascular activity. (**A–E**) Number [n/ml] of blood lymphocytes (**A**), monocytes (**B**), neutrophils (**C**), eosinophils (**D**) and basophils (**E**). (**F**) Plasma interleukin (IL)-6 concentrations [pg/ml]. (**G**) Plasma cortisol concentrations [ng/ml], as readout for hypothalamic–pituitary–adrenal (HPA) axis activity. (H) Salivary α-amylase concentrations [U/ml], as readout for sympathetic nervous system (SNS) activity. (**I**, **J**) Heart rate (HR, [bpm]; **I**) and mean arterial pressure (MAP, [mmHg]; **J**), as readouts for cardiovascular activity. Data are presented as mean + SEM including individual values.

PBMCs isolated from VACs secreted significantly more proinflammatory IL-6 when cultured ex vivo under basal conditions (Fig. 2A; *P* < 0.001) or in the presence of the T cell-specific mitogen ConA (1:10 dilution of the supernatants; *P* < 0.001; Fig. 2B; 1:200 dilution of the supernatants; *P* < 0.001; data not shown), both compared with respective noVACs. Moreover, ex vivo cultured PBMCs from VACs vs. noVACs released more antiinflammatory IL-10, both under basal (Fig. 2D; *P* = 0.007) and ConA (Fig. 2E; *P* < 0.001) conditions. Importantly, basal and ConA-induced PBMC ex vivo IL-6 (basal: *P* < 0.001; ConA: *P* < 0.001) and IL-10 (basal (trend): *P* = 0.056; ConA: *P* = 0.007) secretion were still increased in VACs vs. noVACs when individuals reporting a prior SARS-CoV-2 infection were excluded from the analysis (data not shown). Of note, ex vivo IL-6 (Fig. 2C) and IL-10 (Fig. 2F) secretion in the presence of LPS were not different between the groups. Moreover, blood lymphocyte (Fig. 3A), neutrophil (Fig. 3C), eosinophil (Fig. 3D), and basophil (Fig. 3E) counts, as well as plasma IL-6 concentrations (Fig. 3F) were not affected by the vaccination status. Prior COVID-19 vaccination only by trend increased monocyte (Fig. 3B, P =  0.051) counts compared with respective non-vaccinated participants, with statistical analysis employing ANCOVA testing with “presence of an own pet during early life as covariate” revealing even a significantly increase in blood monocyte counts in the VAC vs. noVAC group (*P* = 0.018).

### Mental and physical health parameters (Tab. 1)

Neither physical complaints nor any mood differences or stress-related symptoms were detected between the experimental groups employing validated questionnaires (Tab. 1; i.e., SCID-I: Structured Clinical Interview for DSM-IV Disorders; STAI-S: State-(Trait-) Anxiety-Inventory; BL: List of complaints for quantitative analysis of current bodily and general complaints; MDBF: Multidimensional Mood State Questionnaire).

**Table 1:** Summary of the results generated by the questionnaires employed in the present study.

Parameter	Not Vaccinated (noVAC; *N* = 24)	Vaccinated (VAC; *N* = 15)	*P*-value (*t*-test; chi^2^)
STAI-S	Mean ± SEM	Mean ± SEM	
Before TSST	35.21 ± 1.49	33.13 ± 1.52	0.360
BL	Mean ± SEM	Mean ± SEM	
Complaints	6.17 ± 1.47	5.00 ± 1.06	0.573
MDBF-A	Mean ± SEM	Mean ± SEM	
Mood	17.54 ± 0.62	18.40 ± 0.42	0.322
Alertness	17.04 ± 0.55	16.67 ± 0.56	0.653
Rest	16.71 ± 0.58	16.73 ± 0.51	0.976
SCID-I (Telephone screening)	No/Unclear/Yes in %	No/Unclear/Yes in %	
Alcohol (Times with more than 5 drinks at one occasion?)	92/0/8	80/0/20	0.289
Drugs (Ever taken?)	92/4/4	80/0/20	0.220
Pharmaceuticals (Felt dependent on or took more than prescribed?)	100/0/0	100/0/0	n.a.
Panic attacks (Ever experienced?)	96/0/4	100/0/0	0.423
Agoraphobia (Ever experienced?)	100/0/0	100/0/0	n.a.
Social anxiety (Ever experienced?)	100/0/0	100/0/0	n.a.
General anxiety (Ever experienced?)	100/0/0	100/0/0	n.a.
Compulsive thoughts (Ever experienced?)	100/0/0	100/0/0	n.a.
Compulsive acts (Ever experienced?)	100/0/0	100/0/0	n.a.
Particularly nervous or anxious (During last 6 months?)	100/0/0	100/0/0	n.a.
Extraordinarily lean (Ever mentioned by others?)	96/0/4	100/0/0	0.423
Binge eating (Ever occurred?)	100/0/0	100/0/0	n.a.

Depicted is the mean ± SEM or the percentage of participants vaccinated (VAC) vs. non-vaccinated against COVID-19 (noVAC), respectively, per group and the *p*-value provided by statistical analysis using either *t*-test or chi^2^ test.

BL, list of complaints for quantitative analysis of current bodily and general complaints; MDBF, multidimensional mood state questionnaire; STAI-S, state(-Trait-) Anxiety-Inventory; SCID-I, structured clinical interview for DSM-IV Disorders; n.a., not assessed.

### Physiological parameters (Fig. 3G–J)

Plasma cortisol concentrations (Fig. 3G), salivary α-amylase concentrations (Fig. 3H), heart rate (HR, Fig. 3I) and mean arterial pressure (MAP; Fig. 3J) were comparable between participants of the VAC and noVAC group.

## Discussion

Available data suggest COVID-19 vaccination to induce RBD-specific T cell responses and not to facilitate T cell (re)activity in general. However, the data presented in this manuscript clearly indicate that isolated PBMCs from individuals vaccinated against COVID-19 show a pronounced increase in basal and ConA-induced ex vivo proinflammatory IL-6 secretion, and a less pronounced increase in basal and ConA-induced antiinflammatory IL-10 secretion, both in comparison with non-vaccinated individuals, while LPS-induced ex vivo IL-6 and IL-10 secretions were not affected by vaccination status, as were plasma IL-6 concentrations, complete blood counts as well as physical and mental health status.

The experimental groups (VAC vs. noVAC) of the present study were statistically comparable with respect to most socioeconomic parameters assessed, except the “professional situation” and “high income”. As medical students, who were also enrolled in the present study, were practically supporting the Ulm University Medical Center during the early difficult times of the COVID-19 pandemic with direct physical contact to potentially infected patients, they were one of the first individuals receiving a vaccination offer. This might explain why more study participants in the noVAC vs. VAC group reported “high income” and “full-time employment”, while more participants in the VAC vs. noVAC group reported “in training”. Of note, the VAC and noVAC group further differed significantly with respect to animal (i.e. pet and/or farm animals) contact during early life (i.e., until the age of fifteen) and also at present. This is of particular importance in the context of the current study, and as we have shown earlier that individuals raised (i.e. until the age of fifteen) in a rural environment with regular contact to farm animals show a less pronounced stress-induced inflammatory in vivo and ex vitro response relative to individuals raised in an urban environment with no regular contact to pets^9^. Therefore, statistical ANCOVA testing with “own pet present” during early life or at present as covariates was employed to successfully confirm all significant vaccination effects revealed between the VAC and noVAC group by Student’s *t*-tests in the present study.

Strikingly, the PBMCs isolated from VACs secreted significantly more proinflammatory IL-6 and IL-10 when cultured ex vivo under basal conditions or in the presence of the T cell-specific mitogen ConA, both compared with respective noVACs. As the ex vivo IL-10 release was increased as a consequence of COVID-19 vaccinations by factor ~ 2–3 under both basal and ConA conditions, while the respective IL-6 release was increased by factor ~ 600 under basal conditions and by factor ~ 6000 under ConA conditions, these findings suggest a strong and SARS-CoV-2-unspecific proinflammatory reprogramming of the T cell compartment by COVID-19 vaccinations. In argument for this ex vivo vaccination effect to specifically affect the adaptive T cell and not the innate myeloid immune cell compartment, ex vivo IL-6 and IL-10 secretion in the presence of LPS were not different between the groups. Interestingly, this vaccination-induced pro-inflammatory shift was mainly detectable under ex vivo and to a much lesser extent under in vivo conditions, as blood lymphocyte, neutrophil, eosinophil, and basophil counts, as well as plasma IL-6 concentrations were not affected by the vaccination status. One possible explanation for the discrepancy between increased basal ex vivo IL-6 secretion but unaffected basal plasma IL-6 levels could be that COVID-19 vaccines only “prime” systemic PBMCs and that a second hit, as for instance the isolation process or ex vivo culturing, is required to fully activate these cells. Of interest in this context might also be recent own preclinical data indicating that stress-induced changes in plasma cytokine levels are rather mediated by bone marrow leucocytes than by systemic immune cells^10^. Prior COVID-19 vaccination only by trend increased monocyte counts compared with respective non-vaccinated participants, suggesting at least a mild in vivo effect on systemic innate immunity. In support of the latter, statistical analysis employing ANCOVA testing with “presence of an own pet during early life as covariate” revealed even a significantly increase in blood monocyte counts in the VAC vs. noVAC group.

Although a more detailed investigation of this phenomenon with longer time periods between vaccination and ex vivo PBMC stimulation is critically required, a lack of correlation of the time period between the 1st, 2nd and 3rd vaccination and the ex vivo IL-6 secretion from isolated PBMCs under ConA conditions (1:200 dilution of supernatants) supports the hypothesis that the here revealed vaccination effect is a non-transient one.

In agreement with the hypothesis that COVID-19 vaccinations mainly affect ex vivo and not in vivo immune cell (re)activity and plasma IL-6 levels, we did neither reveal any physical complaints nor any mood differences or stress-related symptoms between the experimental groups employing validated questionnaires. Of note, many stress-associated mental disorders are accompanied by an over-reactive immune system and chronic low-grade inflammation^11,12^. Moreover, prospective human and mechanistic animal studies strengthen the idea that an exaggerated immune (re)activity plays a role in the development of mental disorders^13,14^. However, as individual differences in IL-6 secretion from ex vivo-stimulated immune cells predict susceptibility versus resilience to a subsequently applied repeated social stressor in mice^15^, long-term studies assessing the risk of COVID-19 vaccinated individuals for the development of stress-related disorders in response to severe and/or chronic psychosocial burden are urgently needed. Against any negative physiological in vivo consequence of COVID-19 vaccinations argue also our findings that plasma cortisol concentrations, as main readout for hypothalamic–pituitary–adrenal (HPA) axis activity, salivary α-amylase concentrations, as readout for sympathetic nervous system (SNS) activity, as well as HR and MAP, as readouts for cardiovascular activity, are comparable between participants of the VAC and noVAC group. Our study also has some limitations that warrant consideration. For instance, although we had the permission from our ethics committee to retrospectively contact our participants again and ask for officially confirmed SARS-CoV-2 infections, whether and when they have been vaccinated against COVID-19, and which type of vaccine they have received, we were not allowed to ask for previous immunizations with vaccines for other pathogens like e.g. Influenza or Hepatitis B Virus. Future studies, thus, need to investigate, if vaccines against other viruses and germs induce similar ex vivo effects on isolated PBMCs. Based on previous studies^9,11,16^, the sample size of the current study was initially calculated to be sufficient for investigating possible differences in the acute TSST-induced immune activation between URBANs raised in the presence vs. absence of pets, and not to investigate the effects of SARS-CoV-2 vaccination on in vivo and ex vivo inflammation markers. Thus, the results of the current study need to be interpreted with caution and confirmed in larger studies, allowing further to assess whether the inflammatory status at the experimental day is dependent on the time elapsed since the 1st/2nd/last COVID-19 vaccination and/or the type of vaccine received. Despite these limitations, we believe that the findings of the present study are of relevance for all studies assessing ex vivo PBMC functionality in both SARS-CoV-2 vaccinated and non-vaccinated participants.

Together, the findings of the current study support the hypothesis that COVID-19 vaccinations unspecifically exaggerate blood adaptive T cell ex vivo (re)activity and, to a lesser extent, in vivo monocyte counts, independent of the desired and specific response towards the RBD of the SARS-CoV-2 spike protein. These findings are of general interest for the scientific community, as they highlight the COVID-19 vaccination status of participants/patients to be considered as critical co-variate, at least for all clinical studies collecting ex vivo PBMC data before and during the COVID-19 pandemic.

## Methods

### Recruiting

This study was approved by the Ethics Committee of Ulm University and is registered at the DRKS (German Clinical Trials Register, ID DRKS00016022). We confirm that all research was performed in accordance with relevant guidelines/regulations and in accordance with the Declaration of Helsinki. A commuting accident insurance was installed for participating volunteers. Experimenters were covered by the employer’s public liability insurance. As this study was designed as a follow up study of^9^, we decided to only include men in the current study. For recruitment a flyer was designed asking for healthy male participants between 18 and 40 years of age who grew up (until the age of 15) in a city with more than 40.000 residents either in the absence of (*n* = 20) or presence (*n* = 20) of own pets (i.e., dog or cat at least during 5 out of the first 15 years). Interested participants were then called, and those who turned out to be physically (asked whether they suffer from chronic physical disorders) and emotionally healthy (Structured Clinical Interview for DSM-IV Disorders, SCID-I, telephone screening), non-regular-smoking, caucasian, non-drug taking (NSAID, cannabis, etc.), non-traumatized (during early life, adolescence and adulthood), non-acutely (within the last 6 months) bereaved or divorced and had a BMI between 20 and 30, were invited to participate in the present study. A total of 64 subjects were screened for this study. 24 individuals were rejected from participation in the study, as they either did not match the above mentioned criteria (not physically healthy: n = 7; not emotionally healthy: n = 2; grew up in a city with less than 40.000 inhabitants: n = 3; parameter “pet” not sufficiently met: n = 2; BMI too high: n = 2; older than 40 years: n = 3) or quitted participation prior to the experimental day (n = 5). For the actual experiment all participants were asked to abstain from caffeine, any kind of drugs (e.g. analgesics, sleep-inducing drugs, dietary supplements), exercise, alcohol and nicotine for a minimum of 3 days. Furthermore, participants were told to sleep at least 8 h during the night before the experiment and to drink at least 1 l of water on the experimental day itself. In cases of unforeseen illness, test persons were told to delay the experiment. Data were collected between July 2020 and April 2022. Of particular importance for the current study, all participants were retrospectively contacted to assess whether and when they have been vaccinated against COVID-19, which vaccine they have received and if they got infected with SARS-CoV-2 prior to the experimental day (Fig. 1). Of note, 4 participants in the noVAC group could not be contacted retrospectively and, therefore, the vaccination and infection status could not be ascertained (i.e., are not visuablized in Fig. 1). However, as the experimental day for these four individuals was prior to the release of the first SARS-CoV-2 vaccine, they were assigned to the noVAC group and included in Figs. 2 and 3, but were excluded from the statistical analysis of ex vivo data considering only non-SARS-CoV-2-infected individuals. If participants only reported the month of vaccination (8 noVAC paricipants; 3 VAC participants)/ infection (6 noVAC participants; 2 VAC participants), the 1st day of the month was considered as date of vaccination/ infection in Fig. 1. Moreover, one participant in the noVAC group was still unvaccinated at the time point of manuscript publication.

### Experimental procedure

On the test day itself, participants were told to arrive at the laboratory at 10 a.m. Only if no signs of illness were reported, the venous catheter (non-dominant arm), as well as the blood pressure and RR analyzer (dominant arm) as well as the ECG chest belt were placed (− 60 min) [*in a room adjacent to the TSST room*]. Immediately prior to catheterization, all participants were informed about possible side effects of the catheterization [*and TSST procedure*] by the PIs and gave informed consent to participate in the current study, while afterwards sociodemographic features and basal physical and emotional health statuses of the participants were assessed, employing validated questionnaires (List of complaints for quantitative analysis of current bodily and general complaints (BL); State-(Trait-)Anxiety-Inventory (STAI-S); Multidimensional Mood State Questionnaire (MDBF)). Additionally, it was assessed whether the participants were born naturally or via cesarean (C-section). At the -5 min time point [*as well as 5, 15, 60, 90, 120 and 150 min after the TSST*], systolic (SBP) and diastolic (DBP) blood pressure were assessed for calculation of MAP, blood was drawn in ethylenediaminetetraacetic acid- (EDTA) and lithium heparin-coated monovettes for collection of plasma and isolation of total [*and CD4*^+^] peripheral blood mononuclear cells (PBMCs), respectively. In addition, blood was drawn in EDTA-coated monovettes at the − 5 min time point [*and 5, 15, 150 min after the TSST*] for analysis of complete blood counts, while saliva samples were collected for determination of α-amylase concentration at the -5 min time point [*and 5, 15, 60, 90, 120 and 150 min after the TSST*]. Heart rate (HR) was assessed continuously between − 5 [*and 150 min*], [as well as *HR variability (HRV)*]. [*After the 5th blood draw (90 min), STAI-S and MDBF were used again to assess subjective strain induced by the TSST procedure. After the 7th blood draw (150 min) the catheter was removed and mental health status (Hospital Anxiety and Depression Scale—German Version, HADS-D; SCID-I (affective part)), early life (Childhood Experience of Care and Abuse Questionnaire, CECA-Q; Childhood Trauma Questionnaire, CTQ) and perceived life stress (Perceived Stress Scale-4, PSS-4) were assessed using validated questionnaires.*]. All samples were treated using the same materials, in the same lab, under the same conditions and by the same experimenter. One participant in the noVAC group had to be removed from all analyses in the current study, as the venous catheter did not work correctly. As repeated attempts to correct this problem might have caused psychosocial stress load for the participant, he was also excluded from analysis of physical and mood health status employing questionnaires. One participant in the noVAC group had to be excluded only from experiments using isolated PBMCs due to problems with the centrifuge on the experimental day. One participant of the noVAC group had to be excluded from the analysis of the item “physical activity”, as the indicated time was unrealistic (i.e. 3600 min/week).

### Blood pressure and heart rate

Systolic and diastolic blood pressures were determined at the − 5 min time point prior to the TSST [*and 5, 15, 60, 90, 120 and 150 min after the TSST*], using a digital brachial blood pressure monitor (Boso Medicus Control, Bosch + Sohn GmbH und Co. KG, Jungingen, Germany), while heart rate of the participants was continuously assessed, using a single-channel ECG (sampling rate 1000 Hz) using a Bittium Faros™ 180 device (Bittium Corp., Oulu, Finland). The cuff placed around the dominant arm at the − 60 min time point [*stayed in place until the last measurement was performed at the 150 min time point; the connection between the cuff and the device was released after each measurement*]. During assessment of the blood pressure the participant was sitting on a chair, placing the arm in a slightly bent position on a table. Systolic and diastolic blood pressures were used for the calculation of MAP according to the formula: DBP+ (SBP-DBP)/3.

### Blood draw

Blood (7.5 ml at each time point) was collected from an indwelling venous catheter in the non-dominant arm (inserted at − 60 min) at the − 5 min time point prior to the TSST [*and 5, 15, 60, 90, 120 and 150 min after the TSST*] into chilled EDTA-coated monovettes. The latter were centrifuged (1000 g/15 min, 4 °C) immediately after each blood draw and plasma was aliquoted and stored at − 80 °C until further processing. Moreover, another 7.5 ml blood were collected via the indwelling venous catheter at the − 5 min time point prior to the TSST [*and 5, 15 and 150 min after the TSST*] into chilled EDTA-coated monovettes for analyses of complete blood counts at the Department of Clinical Chemistry at the Ulm University Medical Centre (Ulm, Germany). In addition, 9 ml of blood were collected via the indwelling venous catheter at the − 5 min time point prior to the TSST [*and 5, 15, 60, 90, 120 and 150 min after the TSST*] into lithium-heparin-coated monovettes and stored on ice until blood from all time points was drawn. Afterwards total [*and CD4*^+^] PBMCs were isolated and used for ex vivo culturing experiments and molecular analysis of FoxP3 expression, respectively.

### PBMC isolation and stimulation

Nine ml blood were transferred from lithium-heparin-coated monovettes into Leucosep™ tubes (Greiner Bio-One GmbH, Frickenhausen, Germany), which were prepared beforehand with Ficoll® Paque (GE Healthcare Life Sciences, Freiburg, Germany) according to the manufacturer’s instructions. The remaining volume was filled up to 50 ml with PBS and then centrifuged for 10 min at room temperature (1000 g, no brake). The buffy coat layer containing PBMCs was transferred into another 50 ml Falcon® tube and washed with RPMI medium containing 10% fetal calf serum (FCS) and 1% penicillin/streptomycin (323 g, 10 min, room temperature). The number of viable (trypan blue) cells was then determined using an automated cell counter (TC20™ Automated Cell Counter, BIO-RAD Laboratories, Munich, Germany), before cells were centrifuged again (323 g, 10 min, room temperature) and adjusted to a final concentration of 2.5 × 10^6^ cells/ml. 2.5 × 10^5^ cells were then cultured in 96-well plates, either under basal conditions (100 µl RPMI were added to a final volume of 200 µl per well) or in the presence of concanavalin A (ConA; final concentration in 200 µl volume was 2.5 µg/ml) or lipopolysaccharide (LPS; final concentration in 200 µl volume was 1 µg/ml) at 37 °C and 5% CO_2_ for 24 h. Supernatants were collected afterwards and stored at − 80 °C until further analysis.

### Enzyme-linked immunosorbent assay (ELISA)

Plasma samples were analysed using commercially available ELISA kits for interleukin (IL)-6 (Quantikine HS ELISA, R&D Systems Europe, Ltd.; lowest standard 0.16 pg/ml) and cortisol (IBL International, Hamburg, Germany; lowest standard 20 ng/ml), respectively, according to the manufacturers’ instructions. Supernatants from PBMC ex vivo stimulations were analysed using commercially available ELISA Kits (Human DuoSet ELISA, 5 Plate, R&D Systems Europe, Ltd) for IL-6 (lowest standard of 9.38 pg/ml; basal, LPS and ConA wells) and IL-10 (lowest standard of 31.3 pg/ml; basal and ConA wells) according to the manufacturer’s instructions.

### Determination of salivary α-amylase concentrations

Salivary α-amylase as a surrogate marker of sympathetic nervous system activity was measured as described earlier^17^. In detail, saliva was processed on a FLUENT liquid handling system (Tecan, Crailsheim, Germany). Saliva was diluted at 1:625 with ultrapure water by the liquid handling system. Twenty microliters of diluted saliva and standard were then transferred into 96-well polystyrol microplates (Roth, Karlsruhe, Germany). Standard was prepared from “Calibrator f.a.s.” solution (Roche Diagnostics, Mannheim, Germany) with concentrations of 326, 163, 81.5, 40.75, 20.38, 10.19, and 5.01 U/l alpha-amylase, respectively, and ultrapure water as zero standard. Afterwards, 50 µl of substrate reagent (α-amylase CC FS; DiaSys Diagnostic Systems GmbH, Holzheim, Germany) was pipetted into each well. The microplate containing sample and substrate was then heated to 37 °C in a Thermomixer (Eppendorf, Hamburg, Germany). Immediately afterwards, a first interference measurement was obtained at a wavelength of 405 nm using a standard absorbance reader (Infinite M200, Tecan, Crailsheim, Germany). The plate was then incubated for another 5 min at 37 °C, before a second measurement at 405 nm was taken. Increases of absorbance in samples were transformed to α-amylase concentrations using a linear regression computed against the standard curve on each microplate. Inter- and intra-assay variation was below 10%.

### Statistics

For statistical analysis and graphical illustrations of immune and physiological parameters, as well as the creation of Fig. 1, GraphPad Prism (version 9.3.1, GraphPad Software, LCC) was used. Parametric statistics was applied to all datasets. Extreme outliers were identified using Grubbs’ test and excluded from further analysis. Data sets were subsequently analyzed using two-tailed Student's *t-test* (one factor, two independent samples) or two-tailed Student's *t-test* with Welch's correction, when appropriate. For statistical analysis of socioeconomic as well as mental and physical health parameters, the software package IBM SPSS statistics (version 28.0.1.0; IBM Corporation, Armonk, NY, United States) was used. Data sets were thereby analyzed using chi^2^ test (nominal scaled variable) or parametric Student’s *t*-test (ratio scaled variables; one factor, two independent samples). Data in Fig. 1 are presented as boxplots including the median (line), the 25th and 75th percentile, as well as the minimum/maximum value (whiskers) and the individual datapoints. Data in Figs. 2 and 3 are presented as mean + SEM including individual data points. The level of significance was set at *P* ≤ 0.050. To exclude that the significant and by-trend effects of prior COVID-19 vaccination on ex vivo and in vivo immune system activation revealed by Student’s *t*-tests (GraphPad Prism) is mediated by the regular contact with own pets during early upbringing and at present, statistical ANCOVA testing with “own pet present” during early life or at present as covariates was done using the software package IBM SPSS statistics (version 28.0.1.0; IBM Corporation, Armonk, NY, United States) to validate all significant vaccination effects revealed by Student’s *t*-tests using GraphPad Prism.

## Supplementary Information

Supplementary Table S1.

Supplementary Table S2.

## Data Availability

The datasets generated in the current study are available from the corresponding author on request.
