# Supplementary material for: COVID-19 vaccination exacerbates ex vivo IL-6 release from isolated PBMCs
Source: Sci Rep. 2023 Jun 12;13:9496. doi: 10.1038/s41598-023-35731-2 (PMC10261110; doi:10.1038/s41598-023-35731-2)
Supplement: Supplementary file 1 — Supplementary Table S1. [file 41598_2023_35731_MOESM1_ESM.pdf]

<u>**SUPPLEMENTARY TABLES AND SUPPLEMENTARY TABLE LEGENDS**</u>

Tab. S1 Sociodemographic features of experimental groups.

**Parameter**	**Not Vaccinated (noVAC; *N* = 24) Mean ± SEM or %**	**Vaccinated (VAC; *N* = 15) Mean ± SEM or %**	***P*-value (*t*-test; chi²)**
**Age (years)**	25.88 ± 0.96	24.13 ± 1.21	0.226
**Height (cm)**	181.08 ± 1.08	184.93 ± 1.89	0.065
**Weight (kg)**	79.96 ± 2.07	81.20 ± 2.86	0.722
**BMI (kg/m²)**	24.37 ± 0.56	23.79 ± 0.89	0.563
**Marital status**			0.423
Married	4%	0%	
Not married	96%	100%	
**Relationship**			0.459
Short-term single	17%	40%	
Long-term single	29%	13%	
Alternating partners	8%	7%	
Long-term relationship (married)	4%	0%	
Long-term relationship (unmarried)	42%	40%	
**Children**			0.423
Yes	4%	0%	
No	96%	100%	
**Children in same household**	0	0	
**Adults in same household**	1 ± 0.3	1.9 ± 0.7	0.166
**Education**			0.385
Secondary school without university entrance diploma	13%	7%	
Secondary school with university entrance diploma	87%	86%	
Other	0%	7%	
**University degree**			0.908
Yes	25%	27 %	

No	75 %	73 %	
**Professional qualification**			0.516
Still in education	21%	33%	
Apprenticeship	21%	7%	
Apprenticeship with master craftsman´s diploma	8%	0%	
University	25%	27%	
Without/other	25%	33%	
**Professional group**			0.040
Unskilled worker	4%	13%	
Skilled worker	4%	0%	
Lower professional group	13%	0%	
Middle professional group	17%	13%	
Higher professional group	8%	0%	
Self-employed	0%	0%	
Never worked before	0%	34%	
Unclear / Student	54%	40%	
**Professional situation**			0.042
Full-time employment	50%	13%	
Part-time employment	13%	0%	
Casual employment	12%	20%	
In training	21%	47%	
Other	4%	20%	
**Net income per month**			0.216
< 400 €	13%	33%	
400 - 1000 €	29%	40%	
1000 - 1500 €	13%	13%	
1500 - 2000 €	8%	7%	
2500 - 3000 €	8%	0%	
3000 - 3500 €	8%	0%	
3500 - 4000 €	0%	7%	

> 4000 €	21%	0%	
**High income (=more than 1500 € net income per month)**	46%	13%	0.036
**Contact with pets and/or farm animals (today)**			0.004
Never	54%	13%	
Sometimes	21%	73%	
Daily/own pet	25%	14%	
**Number of pets (today)**	1.3 ± 0.7	0.3 ± 0.3	0.300
**Species (today)**			0.831
No pet	75%	86%	
Dog	4%	0%	
Cat	8%	7%	
Various mammals	8%	7%	
Mammals & reptiles	5%	0%	
**Contact with pets and/or farm animals (until age 15)**			0.028
Never	42%	7%	
Sometimes	25%	20%	
Daily/own pet	33%	73%	
**Number of pets (until age 15)**	2.8 ± 1.5	1.9 ± 0.5	0.676
**Species (until age 15)**			0.054
No pet	67%	27%	
Dog	4%	33%	
Cat	12%	20%	
Various mammals	13%	20%	
Mammals & reptiles	4%	0%	
**Nutrition**			0.362
Meat-eating	88%	100%	
Vegetarian	4%	0%	
Vegan	8%	0%	
**Taking supplements**	33%	20%	0.368

Protein/Vitamins/Minerals/mixed	3 / 3 / 2 / 0	2 / 0 / 0 / 1	0.175
**Food intolerance**	8%	13%	0.617
Lactose / other	1 / 1	1 / 1	1.000
**Birth & Breast feeding**			
Natural birth	92%	80%	0.289
Was breastfed	92%	100%	0.251
Breastfeed (months)	9.8 ± 1.4	7.4 ± 0.9	0.220
**Physical Activity**			
Everyday activities (min/week)	202 ± 24.3	291 ± 56.4	0.165
Sports (min/week)	206 ± 41.4	228 ± 35.3	0.711
**Alcohol consumption**			0.069
Non-drinking	33%	0%	
Less than once a month	13%	13%	
Once a month	13%	13%	
More than once a month	33%	34%	
Once a week	8%	27%	
Two or three days a week	0%	13%	
Nearly daily	0%	0%	
**Smoking**	0%	13%	0.066

Depicted is the mean ± SEM or the percentage of participants vaccinated (VAC) vs. not vaccinated against COVID-19 (noVAC), respectively, per group and the *p*-value provided by statistical analysis using either *t*-test or chi² test.
