# Supplementary material for: COVID-19 vaccination exacerbates ex vivo IL-6 release from isolated PBMCs
Source: Sci Rep. 2023 Jun 12;13:9496. doi: 10.1038/s41598-023-35731-2 (PMC10261110; doi:10.1038/s41598-023-35731-2)
Supplement: Supplementary file 2 — Supplementary Table S2. [file 41598_2023_35731_MOESM2_ESM.pdf]

Tab. S2 Summary of vaccination and infection status relative to the experimental day.

Participant Nr.	Date of blood draw	1st vaccination: date and type of vaccince		2nd vaccination: date and type of vaccince		3rd vaccination: date and type of vaccince	
26	18.09.21	08.05.21	Biontech	11.06.21	Biontech	04.01.22	Biontech
27	19.09.21	31.03.21	Biontech	12.05.21	Biontech	23.12.21	Biontech
28	09.10.21	06.03.21	Astra Zeneca	30.05.21	Astra Zeneca	23.11.21	Biontech
29	17.10.21	25.05.21	Moderna	06.07.21	Moderna	21.12.21	Moderna
30	04.11.21	05.06.21	Moderna	24.07.21	Moderna	03.01.22	Biontech
31	07.11.21	15.05.21	Biontech	15.06.21	Biontech	15.12.21	Biontech
32	20.11.21	29.05.21	Astra Zeneca	06.07.21	Biontech		
33	21.11.21	08.06.21	Biontech	05.07.21	Biontech	20.12.21	Biontech
34	11.12.21	10.06.21	Biontech	09.07.21	Biontech	03.01.22	Biontech
35	12.12.21	02.06.21	Biontech	07.07.21	Biontech	16.12.21	Biontech
36	17.12.21	20.05.21	Biontech	29.06.21	Biontech	04.12.21	Biontech
37	17.02.22	31.12.20	Biontech	21.01.21	Biontech	10.12.21	Spikevax
38	20.02.22	01.06.21	Johnson&Johnson	15.04.22	Biontech		
39	22.03.22	02.06.21	Johnson&Johnson	22.11.21	Biontech	22.02.22	Biontech
40	21.04.22	15.08.21	Biontech	15.01.22	Moderna		

Depicted is the time point of study enrolment (i.e. date of blood draw), the time points of 1st, 2nd and 3rd vaccination and the respective type of vaccine used of each participant in the VAC group.
